# Measurement of lower-limb asymmetry in professional rugby league: a technical note describing the use of inertial measurement units

**DOI:** 10.7717/peerj.9366

**Published:** 2020-06-23

**Authors:** Daniel J. Glassbrook, Joel T. Fuller, Jacqueline A. Alderson, Tim L.A. Doyle

**Affiliations:** 1 Faculty of Medicine, Health, and Human Sciences, Macquarie University, Sydney, New South Wales, Australia; 2 School of Human Sciences, University of Western Australia, Perth, Western Australia, Australia

**Keywords:** Accelerometer, Acceleration, External mechanical load, Global positioning system, GPS, Injury, Performance, Team sport, Impact

## Abstract

**Background:**

Quantifying lower-limb load and asymmetry during team sport match-play may be important for injury prevention and understanding performance. However, current analysis methods of lower-limb symmetry during match-play employ wearable microtechnology that may not be best suited to the task. A popular microtechnology is global positioning systems (GPS), which are torso worn. The torso location, and the summary workload measures calculated by GPS are not suited to the calculation of lower-limb load. Instead, research grade accelerometers placed directly on the lower-limb may provide better load information than GPS. This study proposes a new technique to quantify external mechanical load, and lower-limb asymmetry during on-field team sport play using inertial measurement units.

**Methods:**

Four professional rugby league players (Age: 23.4  ± 3.1 years; Height: 1.89  ± 0.05 m; Mass: 107.0  ± 12.9 kg) wore two accelerometers, one attached to each foot by the boot laces, during match simulations. Custom Matlab (R2017b, The Mathworks Inc, Natick, MA) code was used to calculate total time, area under the curve (AUC), and percentage of time (%Time) spent in seven acceleration categories (negative to very high, <0 g to >16 g), as well as minimum and maximum acceleration during match simulations. Lower-limb AUC and %Time asymmetry was calculated using the Symmetry Angle Equation, which does not require normalization to a reference leg.

**Results:**

The range of accelerations experienced across all participants on the left and right sides were 15.68–17.53 g, and 16.18–17.69 g, respectively. Clinically significant asymmetry in AUC and %Time was observed for all but one participant, and only in negative (<0 g) and very high accelerations (>16 g). Clinically significant AUC differences in very high accelerations ranged from 19.10%–26.71%. Clinically significant %Time differences in negative accelerations ranged from 12.65%–25.14%, and in very high accelerations from 18.59%–25.30%. All participants experienced the most AUC at very low accelerations (2–4 g), and the least AUC at very high accelerations (165.00–194.00 AU vs. 0.32–3.59 AU). The %Time results indicated that all participants spent the majority of match-play (73.82–92.06%) in extremely low (0–2 g) to low (4–6 g) acceleration intensities, and the least %Time in very high accelerations (0.01%–0.05%).

**Discussion:**

A wearable located on the footwear to measure lower-limb load and asymmetry is feasible to use during rugby league match-play. The location of the sensor on the boot is suited to minimize injury risk occurring from impact to the sensor. This technique is able to quantify external mechanical load and detect inter limb asymmetries during match-play at the source of impact and loading, and is therefore likely to be better than current torso based methods. The results of this study may assist in preparing athletes for match-play, and in preventing injury.

## Introduction

Measuring biomechanical asymmetry of the lower-limb during locomotion is important for athletic populations. Individuals that display inter-limb differences of ∼15% in knee flexor strength, or hip extensor flexibility may be at greater risk of injury than those with less asymmetry (Impellizzeri et al., 2007; Knapik et al., 1991). A recent systematic review also indicates that large inter-limb strength differences may also result in decreased athletic performance, and potentially impact match outcomes (Bishop, Turner & Read, 2018). Measuring inter-limb strength differences may not be useful for the match environment however, as strength is not representative of differences in load experienced at each limb during locomotion. The load experienced by the lower-limb during locomotion, and lower-limb load symmetry can be quantified by external mechanical load. External mechanical load is typically expressed as ground reaction forces (GRF), and can be measured accurately within the laboratory with technology such as force plates and instrumented treadmills (Beckham, Suchomel & Mizuguchi, 2014; Lakomy, 1987). Measuring external mechanical load during prolonged on-field locomotion, and during match-play, is not possible via these methods. Instead, wearable microtechnologies that claim to be able to measure external mechanical load on-field have been developed and may be a useful real-world alternative for measuring external mechanical load during prolonged bouts of activity. Although, research grade technologies such as accelerometers with high sampling rates, may be difficult to access for some sporting populations, and this has likely also contributed to a lack of information about symmetry of lower-limb loading during match-play.

Global Positioning Systems (GPS) are a common form of microtechnology worn on the torso, that can measure team sport workloads. GPS workloads are typically expressed by the distances and speeds covered during training and match-play (Cummins et al., 2013). These summary workload measures are calculated by a unit placed on the torso and are extraneous to the body. As a result, quantifying lower-limb symmetry during match-play with GPS is not possible. Quantifying and understanding asymmetry of lower-limb loading during match-play is likely to be important for load monitoring in professional sport due to its potential relevance to injury prevention. Access to this information could help coaches identify if players are exceeding commonly used asymmetry thresholds (e.g., >15% difference), and subsequently employ strategies (e.g., strength training) to address this imbalance and potentially mitigate injury risk (Soligard et al., 2016). This information may also inform training program design by allowing for tailored drills to accommodate the under- or over-loaded lower-limb. As such, researchers have sought to extend the versatility of GPS units, and utilize in-built accelerometers to calculate external mechanical load variables such as vertical GRFs (Edwards et al., 2018). However, this method tends to exhibit poor reliability (ICC: ≤0.67; CV%: 14–33%), and poor validity when compared to three-dimensional motion analysis and force platform data (Edwards et al., 2018). These poor results can likely be attributed to the torso worn location of GPS units, which allows dissipation of the impact force as it is transmitted through joints and body tissues from the foot to the torso (Derrick, Hamill & Caldwell, 1998; Lucas-Cuevas et al., 2017). Research grade accelerometers placed directly on the lower-limb may overcome these limitations and facilitate the collection of additional and more informative external mechanical load information than GPS (Glassbrook et al., 2019; Patterson, McGrath & Caulfield, 2011; Willy, 2018). The ability to quantify lower-limb load during match-play via these methods may also contribute new information about differences in positional demands within a team. In particular, in sports where positions play on one side of the field, such as soccer or rugby league.

The purpose of this study was to explore the feasibility of a new technique for measuring and interpreting accelerations that occur during team sport using a lower-limb worn accelerometer. We hypothesized that this technique would be feasible and capable of measuring lower-limb asymmetry, as well as differences in external mechanical load between playing positions. This method may compliment current methods of quantifying external mechanical load in team sports and facilitate field-based research that has not previously been possible using current microtechnology-based approaches.

## Methods

### Participants

Four National Rugby League (NRL) players (Age: 23.4 ± 3.1 years; Height: 1.89 ± 0.05 m; Mass: 107.0 ± 12.9 kg) from the same club volunteered to participate in this feasibility study. A total of 98 observations (22–26 observations and 79.6–91.7 min per player) were performed throughout one competitive season. Participants were one winger, one center, and two props. Strength and power characteristics of each player, as collected by club strength and conditioning staff, are presented in Table 1. Body mass and height were not reported to preserve the anonymity of each participant. Participants were free of injury throughout the study. This study was approved by the Macquarie University Human Research Ethics Committee (protocol number: 5201700531). Written informed consent was received from each participant prior to participation.

**Table 1:** Strength and power characteristics.

Participant position	1RM Bench press (kg/BM)^a^	Isometric Mid-Thigh Pull (N/BM)^a^	Countermovement Jump Peak Power (W/BM)^b^	Countermovement Jump Height (cm)^b^
Wing	1.5	58.4	71.8	42.5
Centre	1.3	48.4	85.7	50.3
Prop1	1.2	40.6	66.5	35.2
Prop2	1.4	47.6	55.4	39.0

**Notes.**

kg Kilogram BM Body Mass RM Repetition Maximum N Newtons W Watts cm Centimetres

a Data were collected in late preseason.

b Data were collected in early season.

### Procedures

**Figure 1:**
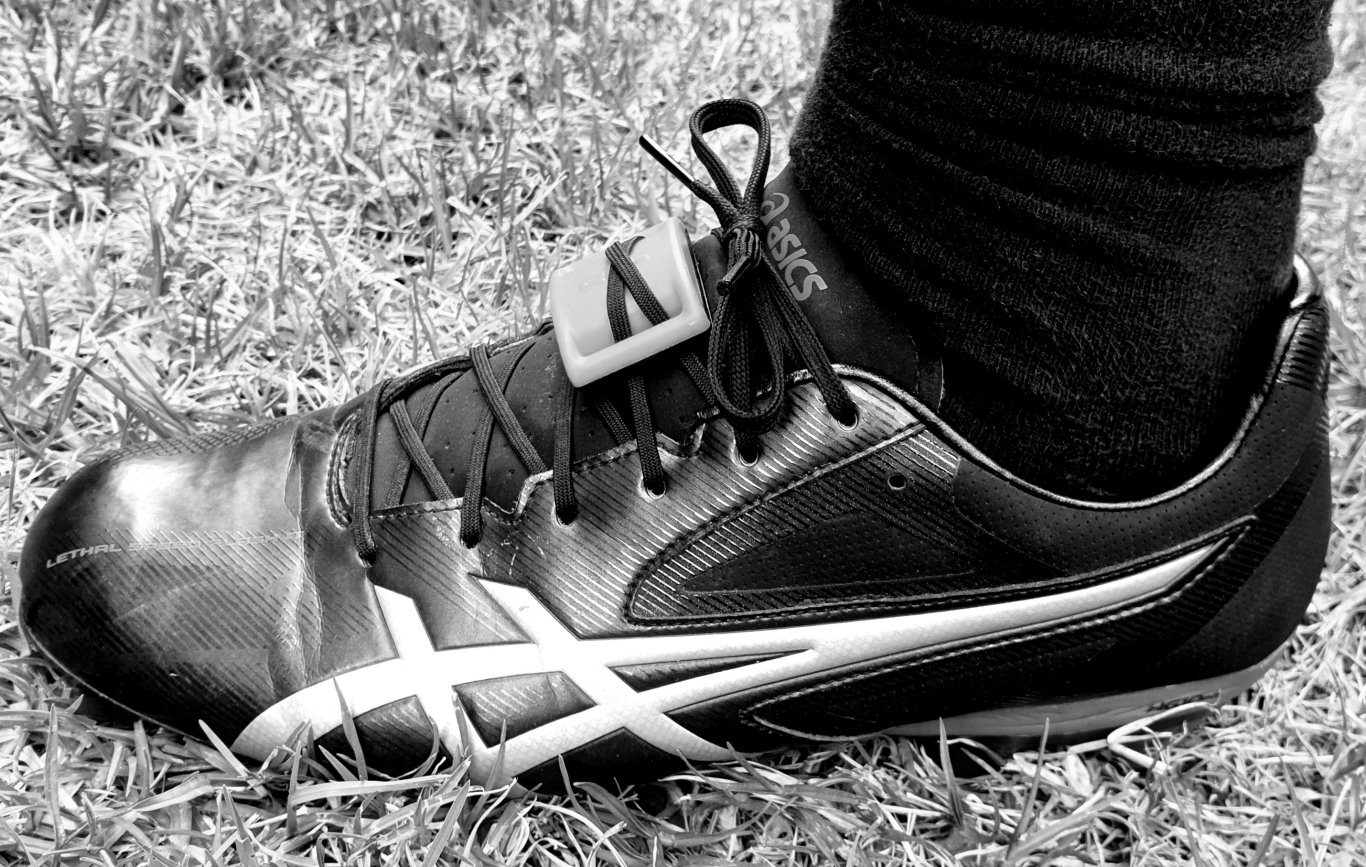
Accelerometer placement on boots.

Participants wore two inertial measurement units (IMU) measuring 40 × 28 × 15 mm and with a mass of 12 g (iMeasureU, Auckland, New Zealand). One accelerometer was attached to each boot using double-sided adhesive tape and by threading the laces through the unit (Fig. 1). The coordinate axes of the accelerometer while on the boot were such that the *X*-axis was orientated in the medial-lateral direction, the *Y*-axis was orientated in the anterior-posterior direction, and the *Z*-axis was orientated in the vertical direction. Applying the sensor to the boot may reduce the risk of injury occurring to the participant if the sensor is impacted, compared to placing the sensor on a bony landmark such as the tibia (Glassbrook et al., 2019). Participants wore the same boots throughout the study. Three-dimensional acceleration data were sampled at 500 Hz and stored directly to the IMU on-board memory. The IMUs measured a maximum of 16 g for each axis and 27 g for the resultant acceleration. Data were collected during 8-minute match simulations over 13 testing sessions. These match simulations are regularly performed in professional rugby league training sessions and are designed to replicate the demands of competition matches by mimicking a typical passage of play in a match. Each participant completed at least 11 sessions, which provided a 6-hour total observation time across all players.

### External mechanical load and asymmetry calculation

The resultant acceleration data for each match simulation were extracted using proprietary software (IMU_Step, Version 1.0, iMeasureU, Auckland, New Zealand). Custom Matlab (R2017b, The Mathworks Inc, Natick, MA) code was then used to convert these data from m/s^2^ to g-force (adjusting for gravity), apply a standard low pass (10 Hz) filter, and calculate total time, area under the curve (AUC) (Arbitrary Units; AU), and percentage of time (%Time) spent within each of seven categories of accelerations: <0 g (negative accelerations), 0–2 g (extremely low intensity), 2–4 g (very low intensity), 4–6 g (low intensity), 6–8 g (moderate intensity), 8–16 g (high intensity), and >16 g (very high intensity) for each accelerometer. The minimum and maximum acceleration value in the negative, and very high intensity categories, respectively, were also extracted. Each limb was analyzed and presented independently, as left and right limb. All four participants indicated their right side as their dominant leg (i.e., based on self-reported preferred kicking leg). Asymmetry between limbs in each category of acceleration for AUC and %Time was calculated using the Symmetry Angle Equation (Zifchock et al., 2008). Previous research has suggested that the symmetry angle equation results in significantly smaller asymmetry percentages (6–7% smaller) than traditional asymmetry equations such as the symmetry index (Błazkiewicz, Wiszomirska & Wit, 2014; Glassbrook et al., 2018). The threshold applied for defining clinically significant asymmetry in this study was 10% (Kvist, 2004; Schmitt, Paterno & Hewett, 2012). In all cases of clinically significant asymmetry, the leg with the highest values was recorded.

### Statistical analysis

Mean ±  standard deviation were used to provide summary information across participants. Mean differences between each limb of each variable were compared across participants for seven categories of accelerations. No inferential statistics were performed because of the small number of participants in this study.

## Results

There was a 100% success rate for data collection with no data loss occurring during any of the observations. No adverse events were reported by any participants as a result of wearing the IMUs during match simulations.

Bilateral individual minimum, and maximum acceleration in negative and very high accelerations, respectively, are presented in Table 2. The range of accelerations experienced across all participants on the left side was 15.68–17.53 g, and on the right side 16.18–17.69 g. The smallest acceleration results for all participants on the left and right sides was observed in Prop1 (left: −0.86 ±  0.06 g; right: −0.83 ± 0.09). The largest acceleration results for all participants on the left and right sides was observed in the wing (left: 18.28 ± 1.17 g; right: 18.41 ±  1.74 g).

**Table 2:** Minimum and maximum acceleration results from left and right sides in negative (<0 g), and very high (>16 g) accelerations.

Side	Participant	<0 g	>16 g
Left	Wing	−0.75 ± 0.10	18.28 ± 1.17
	Centre	−0.77 ± 0.07	16.90 ± 1.81
	Prop1	−0.86 ± 0.06	16.54 ± 0.82
	Prop2	−0.73 ± 0.12	16.89 ± 0.75
Right	Wing	−0.72 ± 0.09	18.41 ± 1.74
	Centre	−0.77 ± 0.12	17.58 ± 1.21
	Prop1	−0.83 ± 0.09	17.01 ± 1.19
	Prop2	−0.74 ± 0.09	17.22 ± 1.44

**Notes.**

g, G-Force. Results presented as mean ± standard deviation.

The results of the symmetry analysis are presented in Table 3. Clinically significant asymmetry (i.e., >10%) in AUC between left and right limbs was observed at very high accelerations in all participants except one, the winger. Clinically significant AUC differences between legs ranged from 19.10%–26.71% for all participants, except the winger, with the right leg consistently providing greater AUC values than the left leg. Clinically significant asymmetry in %Time were observed for three of the participants, the center and both props, in negative and very high accelerations, with the right leg consistently providing greater %Time values than the left leg. Clinically significant %Time differences between legs in negative accelerations ranged from 12.65%–25.14%, and in very high accelerations from 18.59%–25.30% in all participants except the winger.

**Table 3:** Symmetry angle (expressed as percentage difference) for area under the curve (AU) and percentage of time (%min) for left and right sides, in seven categories of accelerations.

Variable	Participant	<0 g	0–2 g	2–4 g	4–6 g	6–8 g	8–16 g	>16 g
AUC	Wing	4.69	1.64	1.29	1.02	1.42	0.03	3.97
	Centre	3.26	0.90	0.07	1.75	1.72	2.10	21.21^a^
	Prop1	3.37	0.48	0.10	0.98	2.67	4.73	26.71^a^
	Prop2	6.10	2.76	2.33	2.84	2.06	6.27	19.10^a^
%Time	Wing	4.17	1.36	1.27	1.04	1.52	0.13	2.70
	Centre	25.09^a^	6.53	0.12	2.03	1.64	1.85	22.25^a^
	Prop1	12.65^a^	2.65	0.03	1.00	2.68	5.30	25.30^a^
	Prop2	25.14^a^	2.06	1.92	2.63	2.30	5.97	18.59^a^

**Notes.**

g G-Force AUC area under the curve %Time percentage of time

a clinically significant asymmetry (>10%).

**Figure 2:**
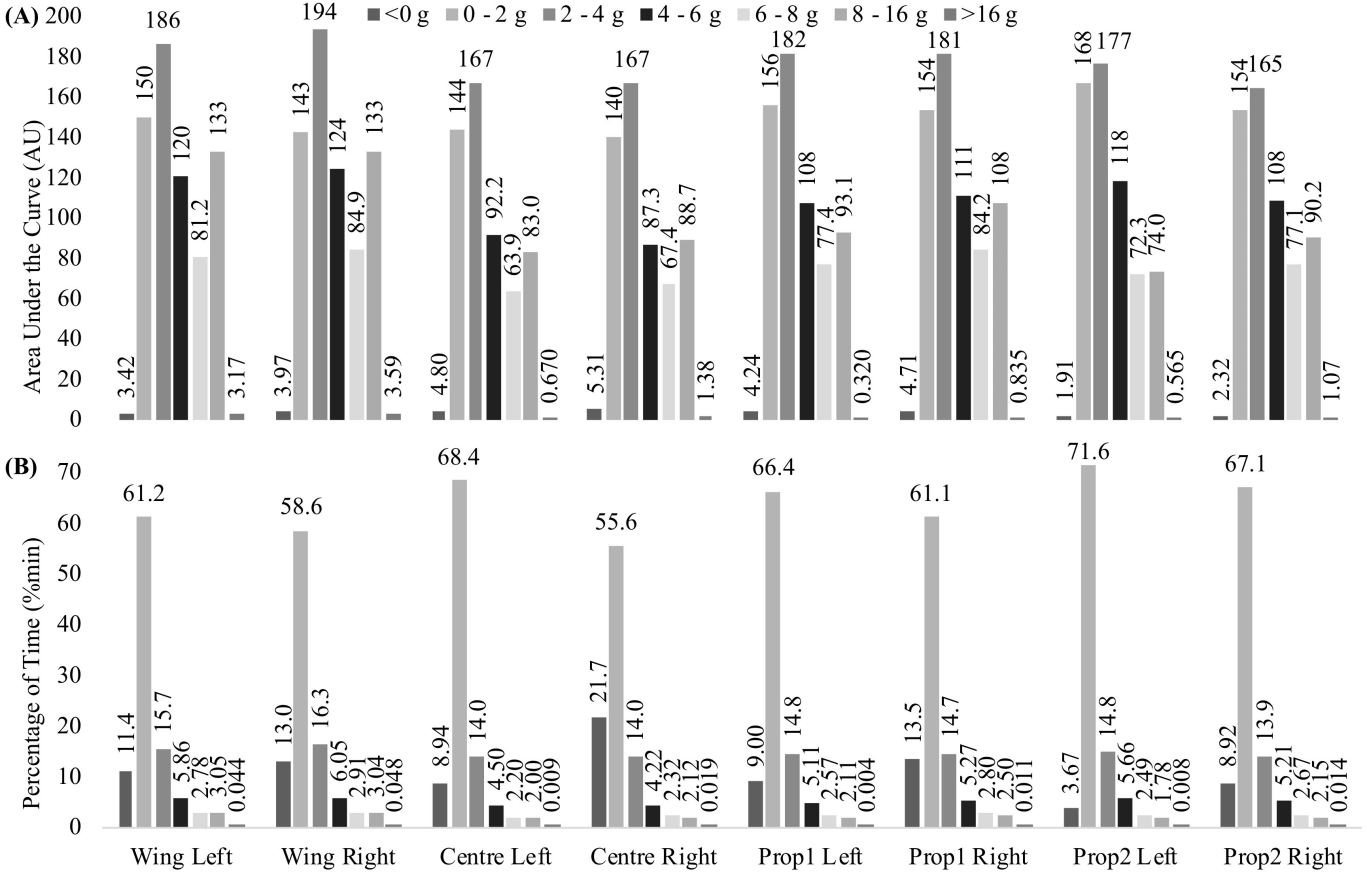
(A) Area under the curve (AU), and (B) percentage of time (%min) results for left and right sides in seven categories of accelerations. g, G Force.

Bilateral individual mean AUC and %Time results, in seven categories of acceleration are presented in Fig. 2. A similar trend in AUC magnitudes were observed across all participants, in that across the seven categories of acceleration, the categories ranked from the highest to lowest AUC magnitudes remained constant. For example, all participants experienced the highest AUC in very low accelerations, followed by extremely low accelerations, and the least in very high accelerations. The AUC data indicated differences in absolute magnitudes between participants across each of the seven categories of acceleration (range: 12.00–167.00% difference). The only time equal AUC results were observed was by the wingers left and right sides in high accelerations (133.00 AU). The acceleration intensity in which all participants experienced the most AUC was the very low accelerations (165.00–194.00 AU) followed by extremely low accelerations (140.00–168.00 AU). Conversely, all participants experienced the least AUC in very high accelerations (0.32–3.59 AU), followed by negative accelerations (1.91–5.31 AU). The %Time data indicated that all participants spent the majority of match-play (73.82–92.06%) in extremely low to low acceleration intensities. In contrast to the AUC results, the acceleration intensity band which all participants spent the most %Time in was extremely low accelerations (55.60%–71.60%). All participants spent the next highest %Time in very low accelerations (14.00%–16.30%), except for the center’s right leg which spent the second largest %Time in negative accelerations (21.70%). All participants spent the least %Time in very high accelerations (0.01%–0.05%), however the second smallest %Time spent in an acceleration category was in high intensity accelerations (1.78%–2.75%) for all participants, except for the winger who spent the second least %Time in moderate accelerations (2.78–2.91%).

## Discussion

The purpose of this study was to explore the feasibility of a new technique for measuring and interpreting accelerations that occur during team sport using a lower-limb worn accelerometer. The technique demonstrated excellent feasibility with no data loss and no adverse events occurring throughout the study. The technique was able to detect differences across player positions and enabled the assessment of asymmetries using two unique variables, AUC and %Time. The main findings were (1) clinically significant asymmetry in AUC was observed only at very high accelerations in all participants except the winger, (2) clinically significant asymmetry in %Time was observed for all participants except the winger in negative and very high accelerations, (3) a similar trend of AUC magnitudes across the seven categories of acceleration was observed across limbs within participants, but there were differences in magnitudes across participants, and (4) all participants spent the majority of match simulations in very low acceleration intensities and a similar amount of time in negative, low, moderate and high acceleration intensities across limbs.

It is not surprising that the results of this study show that each participant spent the majority of the match simulations in extremely and very low-intensity acceleration intensities based on %Time data. Previous research in professional rugby league has demonstrated that up to 78% of the total distance covered during match-play is achieved via low intensity movement, walking or jogging (<12 kph) (Austin & Kelly, 2013; McLellan, Lovell & Gass, 2010; McLellan, Lovell & Gass, 2011). In this regard, %Time measures from foot accelerometers are similar to GPS and provide a valid representation of rugby league match-play but may not be required to supplement the summary workload variables calculated by GPS, in the pursuit of external load monitoring. In contrast, the AUC results display a more uniform distribution of external mechanical load across the low to high acceleration intensity bands than %Time, i.e., the trend in AUC magnitudes (Fig. 2). This suggests that, although most of the time is spent in activities associated with extremely low intensity acceleration, players accumulate meaningful external mechanical load across activities associated with low, moderate, and high acceleration. As a result, the AUC variable may provide more insightful information about the external mechanical load experienced at the lower-limb during team sport match simulations than %Time. Future research in this area with a larger sample size than the current study is warranted to confirm these results.

Other than the negative accelerations that result from being forcibly stopped, by way of collision, negative accelerations commonly occur as a player decelerates and voluntarily stops the body’s momentum, after a high-speed effort such as a sprint (Hewit et al., 2011). To do so, the athlete must slow their movement through eccentric muscle actions when the athlete experiences the impacts associated with the foot contacting the ground (Hewit et al., 2011). A clinically significant greater duration of time spent in negative accelerations on the dominant (i.e., preferred) leg, without asymmetry in AUC, indicates that although the magnitude of force the athlete is exposed to from the ground is similar, the dominant leg is spending longer in contact with the ground. Therefore, the dominant leg is contributing greater eccentric muscle braking action than the non-dominant leg to reduce the body’s velocity. The clinical implications of this observation may guide exercise prescription, and should be investigated further, particularly given the potential for eccentric muscle action to contribute to muscle damage and injury (LaStayo et al., 2003). For example, athletes with clinically significant asymmetry between limbs may benefit from the incorporation of eccentric strength exercises into their training programs, and further program individualization to optimize athletic performance.

The symmetry analysis in this study showed that clinically significant asymmetry in external mechanical loading can present during rugby league match simulations, however, it tends to only appear at the extreme ends of the acceleration intensity categories applied in the present study (i.e., negative and very high intensity). This is important as high acceleration movements are often involved in injury mechanisms (Brown, Brughelli & Hume, 2014; Opar, Williams & Shield, 2012), and clinically relevant asymmetry between limbs above a threshold may be indicative of risk of injury (Impellizzeri et al., 2007; Knapik et al., 1991). Notably, the methods in this study utilized a sensor applied directly to the foot as opposed to torso-mounted GPS units, which are the current industry standard for player workload measurement. We speculate that the foot placement used in the present method is a better placement location for detecting lower-limb asymmetry compared to the torso placement used with GPS.

Clinically significant asymmetry was observed in both AUC and %Time at very high acceleration intensities, but only in %Time for negative accelerations. In all cases, the right limb demonstrated higher values than the left leg, and these results may be explained by leg dominance in each player, and an inherently stronger use of the dominant leg for high intensity movement than the non-dominant leg. This is the principle of laterality, in that humans will choose a preferred side to perform motor tasks, in this case the dominant leg (i.e., preferred kicking leg) (Deborah, Richard & Stephan, 2006). All four participants indicated their right side as their dominant leg. The results show that the right side was exposed to 2-3 more times AUC in very high accelerations compared with the left side, resulting in clinically significant asymmetry (>10% difference). However, the absolute magnitude of the left versus right AUC differences at very high intensity accelerations was very small. As a result, clinically significant, relative asymmetry in AUC within a match is unlikely to be sufficient to cause an immediate injury, unless combined with inappropriate joint or muscle positions (e.g., over stretching a muscle). In contrast, we speculate that, regardless of joint and muscle position, the accumulation of a greater amount of AUC on one side of the body over the course of a season is likely to be relevant to overuse injury risk. This potential application of the AUC method to team sport athlete monitoring should be explored in larger, subsequent studies.

The technique used in this study demonstrated that AUC and %Time were different for the wing position when compared to the center and prop positions. The participant in the wing position demonstrated noticeably larger high intensity AUC compared to the other three participants. The winger also demonstrated noticeably more %Time in negative and very high accelerations than the center and props. The winger also demonstrated no clinically significant asymmetry values, whereas the center and props did. All positions included in this analysis remained on one side of the field for the duration of each match-play simulation, as is customary for each position. These lower-limb load results may be partially attributed to the tactical differences between each position in this study. In rugby league, wingers and center’s run faster and perform longer, and relatively more steady-state bouts of activity compared to props (Austin & Kelly, 2013; McLellan, Lovell & Gass, 2011). The wingers are also typically the fastest player in a rugby league team (Meir et al., 2001). This may explain the greater accelerations in the very high intensity acceleration band for the winger in this study compared to the center and props. As the winger is traveling at higher speeds, they will decelerate over a larger distance, which equates to more negative accelerations than the center and props. Moreover, relative to body mass, the winger was the strongest participant in this study across the bench press and isometric mid-thigh pull (Table 1). This level of strength may be a key factor attributing to the winger’s lack of asymmetry in AUC and %Time. Previous research has shown that absolute strength can influence asymmetry, and stronger athletes tend to demonstrate less asymmetry than weaker athletes in lower body strength tests (Bailey et al., 2015).

## Conclusions

This study identified a highly feasible technique for measuring lower-limb external mechanical load during team sport match-play simulations that is capable of detecting clinically meaningful asymmetry and differences across players. As a result, this technique is likely to be meaningful for athlete monitoring programs, injury prevention and performance enhancement. The differences between sides of the body and between players were detected at high intensities, which highlights the need for wearable technology that is capable of measuring a high acceleration range. An understanding of the nature of the lower-limb accelerations experienced by team sport athletes during match-play, coupled with the knowledge of when injury is most likely to occur, may assist in injury prevention. With this knowledge, high performance staff may be able to better prepare athletes for the demands of match-play and assess an athlete’s readiness to return to play post-injury.

##  Supplemental Information

10.7717/peerj.9366/supp-1 Supplemental Information 1 Raw Data

## Additional Information and Declarations

### Data Availability

The following information was supplied regarding data availability: The raw area under the curve, time, minimum and maximum acceleration data are available in the Supplemental File.
